# Patterns and predictors of sick leave after Covid-19 and long Covid in a national Swedish cohort

**DOI:** 10.1186/s12889-021-11013-2

**Published:** 2021-05-31

**Authors:** Emma Westerlind, Annie Palstam, Katharina S. Sunnerhagen, Hanna C. Persson

**Affiliations:** Department of Clinical Neuroscience, Institute of Neuroscience and Physiology, Sahlgrenska Academy, University of Gothenburg, and Sahlgrenska University Hospital, Per Dubbsgatan 14, 3 tr, 413 45, Gothenburg, Sweden

**Keywords:** Covid-19, SARS-CoV2, Sick leave, Long Covid, Follow-up

## Abstract

**Background:**

The impact of Covid-19 and its long-term consequences is not yet fully understood. Sick leave can be seen as an indicator of health in a working age population, and the present study aimed to investigate sick-leave patterns after Covid-19, and potential factors predicting longer sick leave in hospitalised and non-hospitalised people with Covid-19.

**Methods:**

The present study is a comprehensive national registry-based study in Sweden with a 4-month follow-up. All people who started to receive sickness benefits for Covid-19 during March 1 to August 31, 2020, were included. Predictors of sick leave ≥1 month and long Covid (≥12 weeks) were analysed with logistic regression in the total population and in separate models depending on inpatient care due to Covid-19.

**Results:**

A total of 11,955 people started sick leave for Covid-19 within the inclusion period. The median sick leave was 35 days, 13.3% were on sick leave for long Covid, and 9.0% remained on sick leave for the whole follow-up period. There were 2960 people who received inpatient care due to Covid-19, which was the strongest predictor of longer sick leave. Sick leave the year prior to Covid-19 and older age also predicted longer sick leave. No clear pattern of socioeconomic factors was noted.

**Conclusions:**

A substantial number of people are on sick leave due to Covid-19. Sick leave may be protracted, and sick leave for long Covid is quite common. The severity of Covid-19 (needing inpatient care), prior sick leave, and age all seem to predict the likelihood of longer sick leave. However, no socioeconomic factor could clearly predict longer sick leave, indicating the complexity of this condition. The group needing long sick leave after Covid-19 seems to be heterogeneous, indicating a knowledge gap.

**Supplementary Information:**

The online version contains supplementary material available at 10.1186/s12889-021-11013-2.

## Background

### Aim

The aim of this study was to investigate patterns of sick leave, as well as factors predicting sick leave ≥1 month and sick leave for long Covid in hospitalised as well as non-hospitalised persons with Covid-19, in a comprehensive national population.

The Covid-19 pandemic affects the entire community and the long-term consequences are incalculable for the economy, public health, healthcare, and the health insurance system. Symptoms of Covid-19 vary, as do the severity and extent of functional impairment over time [1–6].

Sick leave can be seen as a complex indicator of well-being in the working-age population. The consequences of Covid-19 for working ability and sick leave are not yet known. A large cohort study comprising more than 1.6 million workers in Spain compared total sick leave in the first trimester of 2020 with that in previous years and found that, overall, sick leave was 116% higher in March 2020, mostly related to infectious and respiratory disease [7]. The availability of paid sick leave varies among countries around the world [8]_,_ and in Sweden the financial compensation for sick leave is tax-funded and comprehensive. Furthermore, in Sweden the pandemic initially brought a rapid increase in sick leave, almost doubling the amount of sick leave during March and April 2020 compared with the previous year, according to the Swedish Social Insurance Agency (SSIA) [9]. The SSIA and the National Board of Health and Welfare in Sweden provide diagnosis-specific guidelines on sick leave and, since June 2020, for Covid-19. However, the guidelines for Covid-19 are vague due to a lack of knowledge of the prognosis of the disease—regardless of their need for inpatient care, an affected person’s ability to work could decline in the aftermath [10].

It has been shown that symptoms related to Covid-19 can have a protracted course requiring intensive medical resources, whether or not the acute phase was critical [11–15]. Long Covid is a term describing this condition after the infection, with long-term symptoms such as fatigue, dyspnoea, pain, and depression [13–16]. The World Health Organization [16] defines long Covid as symptoms beyond 12 weeks. However, the cause, prevalence, duration, and prognosis of the protracted symptoms are still not clear [16].

## Methods

### Settings and study population

The present study is a registry-based study with data from the SSIA, the Swedish National Board of Health and Welfare, and Statistics Sweden, based on the unique Swedish personal identification number. It presents data on sick leave due to Covid-19 that started between March 1 and August 31, 2020, with follow-up for 4 months. A patient has been involved in the study process as a partner in research.

Inclusion criteria for the study population were the following: being registered with sickness benefits due to Covid-19, which were defined as the International Statistical Classification of Diseases (ICD) [17] code U07, starting within the inclusion period. Codes U (U00-U49) are used by WHO for provisional assignment of new diseases of uncertain etiology (Please see this link: https://icd.who.int/browse10/2019/en#/U07.1).

The SSIA is the public authority in Sweden that makes decisions on sick leave and pays sickness benefits, and it provided the study population and sick leave data for the present study. All working people in Sweden are eligible for sickness benefits from the SSIA if deemed to have reduced work ability due to sickness, regardless of citizenship or place of residence. Furthermore, self-employment, parental leave, and unemployment (after previous employment) also entitle one to sickness benefits. The employer provides sick pay during the first 2 weeks of sickness absence; thereafter, the SSIA pays sickness benefits. If a person is unemployed, sickness benefits are provided by the SSIA from the start. In the present study, receiving sickness benefits regardless of amount is defined as sick leave.

The National Board of Health and Welfare provided data on date of death during the study period from the Cause of Death Register, which records all cases of death that have been registered in Sweden. Data from the National Patient Register, which includes all inpatient care in Sweden, were used to investigate hospital stay due to Covid-19.

Statistics Sweden holds registries of all people registered in Sweden. Statistics Sweden provided data on sociodemographic variables to the study.

### Variables

The sick-leave period in the present study includes at least one period of sickness benefits due to Covid-19 diagnosis. Other predefined related diagnoses were merged with the Covid-19 sick leave if the gap of non-registration between sick leaves was ≤2 weeks. The related diagnoses are shown in additional Table 1 and included, for example, unspecified virus infections, fever, and postviral fatigue syndrome, but also a second sick-leave registration for Covid-19 diagnosis. In cases of sick pay provided by the employer, these were also merged with the sick-leave period. The sick-leave period could comprise a maximum of 122 days (4 months of follow-up). For predictive analyses, sick leave was dichotomised into sick leave ≥1 month (≥30 days): yes/no, and sick leave for long Covid (≥ 12 weeks, in line with the WHO definition [16]): yes/no.

Sick leave prior to Covid-19 was defined as either being on sick leave for one period of at least 28 days between March 1, 2019, and the date of first Covid-19 sick leave registration, or being on sick leave at least six times during the same period of time.

The SSIA register includes the employment status for which a person receives the sick leave. The types of employment comprise *employment* (including parental leave, and combined employment and self-employment), *self-employment*, and *unemployment* (including studies).

Educational level was categorised as *primary school* (≤ 9 years), *secondary school* (10–12 years), *short university education* (13–14 years), or *long university education* (≥15 years). The educational level registered in 2019 was used. The income variable was the disposable income for each person during 2019, presented in thousands of Swedish krona SEK (1 Euro = 10.16 SEK, March 4, 2021). Income was categorised in tertiles of low, medium, and high income. Country of birth was presented as *Sweden*, *Nordic countries except for Sweden*, *European countries except for the Nordic countries*, and *Countries outside of Europe*. For marital status in 2020, married and registered partnership were both classified as *married*. Likewise, *divorced* and *widow/widowed* meant a change from either marriage or registered partnership. Inpatient care due to Covid-19 was classified as being registered with a hospital stay of > 1 day with a registration of any of the Covid-19 diagnoses U07. The primary diagnoses are presented in additional Table 2 in the cases where U07 was not registered as the primary diagnosis.

### Statistical methods

The data were processed and analysed using IBM SPSS Statistics 25. Data are presented as number and percentage (%), mean and standard deviation (SD), and median and interquartile range (IQR). The significance level (alpha) was set to 5%. To compare differences between groups, the Mann-Whitney U test and Fisher exact test were used.

To graphically present cumulative incidence of sick leave over time, Kaplan-Meier curves were used. There was no censoring, as cases of death during the study period were treated with a worst-case-scenario approach and were set at sick leave for the maximum number of days, and there was no other loss to follow-up.

Multiple logistic regression was used for predictive analysis. The regression analyses were performed on three separate populations: the total study population, the participants receiving inpatient care for Covid-19, and the participants not receiving inpatient care. Two different dependent variables were used in different models: sick leave ≥1 month and sick leave for long Covid. The independent variables were chosen based on clinical and theoretical reasoning: age, sex, educational level, income, country of birth, sick leave prior to Covid-19, employment status, marital status, and inpatient care due to Covid-19. The results are presented as odds ratio (OR), 95% confidence interval (95% CI), and *p*-value in forest plots. The ordinal or continuous independent variables were tested for multicollinearity using the Spearman correlation test, with values < 0.3 being acceptable. To test the accuracy of the models, receiver operating characteristics (ROC) curves were constructed. An area under the ROC curve > 0.70 indicate acceptable accuracy [18].

## Results

### Study population

The study population consists of 11,955 included participants. Of these, 7983 (66.8%) were registered with sick leave due to” Covid-19, virus detected”, 3949 (33.0%) as” Covid-19, virus undetected”, and 23 (0.2%) as “unspecified Covid-19 diagnosis”. As presented in Tables 1, 2960 (24.8%) received inpatient care due to Covid-19 during the study period. Women comprised 60% of the total study population (*n* = 7129), whereas men were 64% (*n* = 1894) of the participants receiving inpatient care due to Covid-19.

**Table 1:** Characteristics of the participants

	Total	Inpatient care	No inpatient care
Participants, n (%)	11,955 (100.0)	2960 (24.8)	8995 (75.2)
Sex, n (%)			
Men	4826 (40.4)	1894 (64.0)	2932 (32.6)
Women	7129 (59.6)	1066 (36.0)	6063 (67.4)
Age, mean (SD)	48.0 (11.3)	52.0 (9.9)	46.7 (11.4)
Country of birth, n (%)^a^			
Sweden	7545 (63.1)	1558 (52.7)	5987 (66.6)
Nordic countries except for Sweden	271 (2.3)	85 (2.9)	186 (2.1)
European countries except for the Nordic countries	1210 (10.1)	370 (12.5)	840 (9.3)
Countries outside of Europe	2929 (24.5)	944 (31.9)	1976 (22.9)
Educational level, n (%)^b^			
Primary school (≤9 years)	1237 (10.4)	411 (14.0)	826 (9.2)
Secondary school (10–12 years)	5889 (49.6)	1406 (47.9)	4483 (50.2)
Short university education (13–14 years)	1743 (14.7)	466 (15.9)	1277 (14.3)
Long university education (≥15 years)	3995 (25.2)	650 (22.2)	2345 (26.3)
Income, 1000 SEK median (IQR)^c^			
Low income	212 (61)	213 (66)	211 (60)
Medium income	288 (33)	290 (33)	287 (33)
High income	390 (100)	404 (121)	384 (94)
Marital status, n (%)^d^			
Married	5812 (48.8)	1695 (57.8)	4117 (45.8)
Single	3859 (32.4)	671 (22.9)	3188 (35.5)
Divorced	2097 (17.6)	536 (18.3)	1561 (17.4)
Widow/Widower	149 (1.3)	31 (1.1)	118 (1.3)
Sick leave prior to Covid-19, n (%)^e^			
Sick leave ≥28 days	1918 (16.0)	357 (12.1)	1561 (17.4)
Sick leave ≥6 times	26 (0.2)	5 (0.2)	21 (0.2)
Employment status, n (%)^f^			
Employment	11,460 (95.9)	2756 (93.1)	8704 (96.8)
Self-employment	288 (2.4)	105 (3.5)	183 (2.0)
Unemployment	204 (1.7)	99 (3.3)	105 (1.2)

a = 9 missing, b = 91 missing, c = 2 missing, d = 38 missing, e = 13 participants on sick leave both ≥28 days and ≥ 6 times, f = 3 missing. Abbreviations: SD, standard deviation; IQR: interquartile range.

A total of 24 participants (0.2%) died within the study period, all within ≤9 days from the end of the sick-leave period.

### Sick-leave patterns

Of the participants, 1931 (16.2%) were on sick leave for at least 28 d or 6 times the year prior to the Covid-19 infection.

**Fig. 1:**
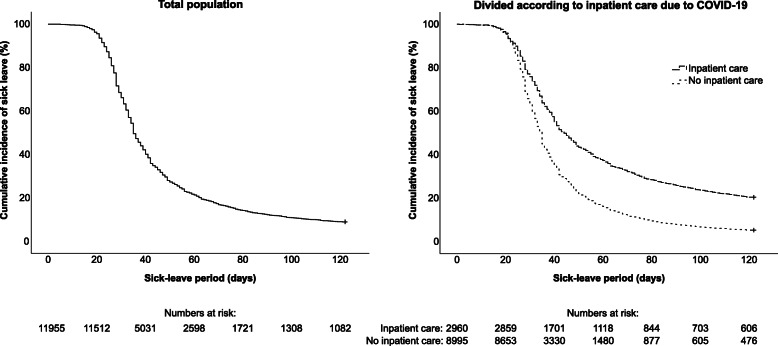
Cumulative incidence of sick leave during the study period. Total study population (left) and divided according to inpatient care due to Covid-19 (right)

The median duration of sick leave due to Covid-19 was 35 days (IQR 26, mean 47.5, SD 29.96). There were 7903 (66.1%) participants on sick leave ≥1 month, and 1073 (9.0%) continued their sick leave during the whole follow-up period, that is, for at least 4 months (Fig. 1).

A total of 1592 (13.3%) were on sick leave for at least 12 weeks, and thus defined as having long Covid. The participants on sick leave for long Covid were significantly older (*p* < 0.001), predominantly men (*p* < 0.001), spent more time on sick leave prior to Covid-19 (*p* < 0.001), and were more likely to have received inpatient care (*p* < 0.001) than the participants not on sick leave for long Covid.

### Predictors of sick leave

**Fig. 2:**
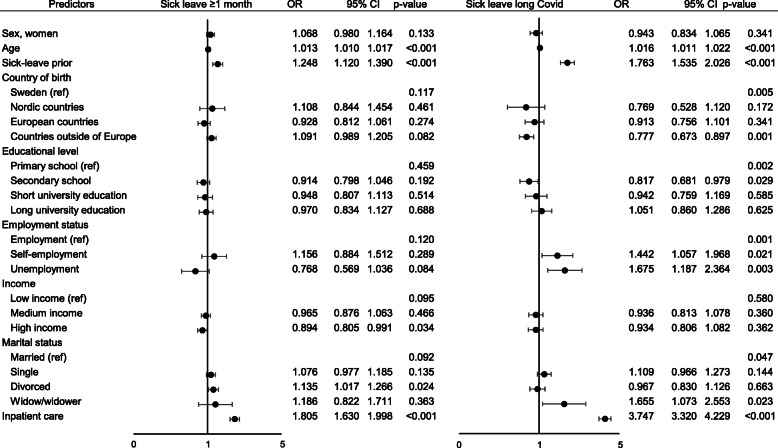
Predictors of sick leave ≥1 month, and long Covid. There were 7813 persons on sick leave ≥1 month, and 4009 < 1 month; area under the ROC curve: 0.586. There were 1574 persons on sick leave for long Covid, and 10,248 not on sick leave for long Covid; area under the ROC curve: 0.692. Abbreviations: OR, odds ratio; CI, confidence interval

Predictors of sick leave ≥1 month, and predictors of sick leave for long Covid, are presented in Fig. 2. Older age, sick leave prior to Covid-19, and inpatient care due to Covid-19 resulted in significantly higher odds for being on sick leave with both outcomes. Different socioeconomic factors were significant for sick leave ≥1 month and sick leave for long Covid, respectively.

**Fig. 3:**
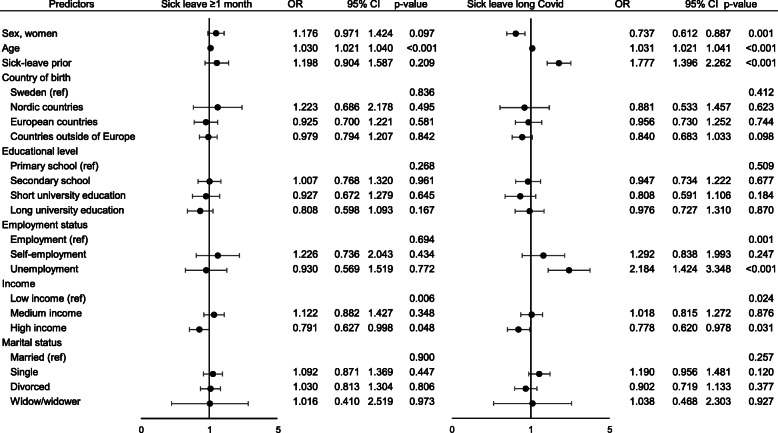
Predictors of sick leave ≥1 month, and long Covid, for participants’ receiving inpatient care. There were 2202 persons on sick leave ≥1 month, and 704 < 1 month; area under the ROC-curve: 0.607. There were 785 persons on sick leave for long Covid, and 2121 not on sick leave for long Covid; area under the ROC-curve: 0.617. Abbreviations: OR, odds ratio; CI, confidence interval

For the participants receiving inpatient care due to Covid-19, older age meant significantly higher odds, and higher income significantly lower odds, of sick leave both ≥1 month and for long Covid (Fig. 3). Other variables differed between the models.

**Fig. 4:**
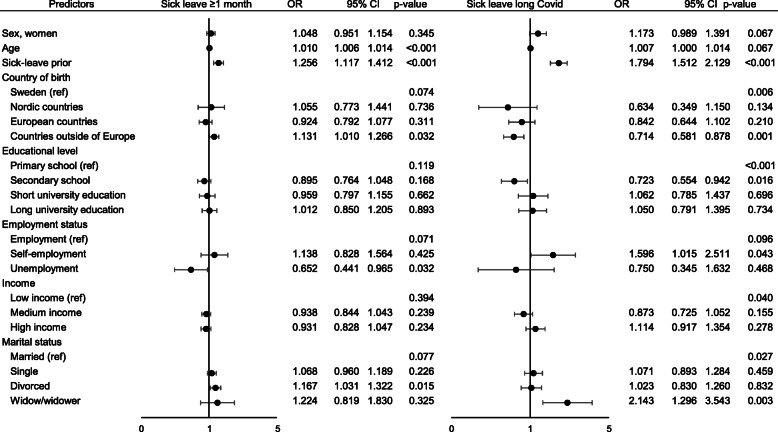
Predictors of sick leave ≥1 month, and long Covid, for participants’ not receiving inpatient care. There were 5611 persons on sick leave ≥1 month, and 3305 < 1 month; area under the ROC-curve: 0.550. There were 789 persons on sick leave for long Covid, and 8127 not on sick leave for long Covid; area under the ROC-curve: 0.609. Abbreviations: OR, odds ratio; CI, confidence interval

For the participants not receiving inpatient care due to Covid-19, sick leave prior to Covid-19 resulted in significantly higher odds of sick leave in both outcomes (Fig. 4). Age, country of birth, employment status, and marital status all gave different odds of sick leave ≥1 month, and sick leave for long Covid, respectively.

## Discussion

In this registry-based national cohort, the median duration of sickness benefits due to Covid-19 was more than 1 month (35 days). Furthermore, more than one out of ten subjects were on sick leave for more than 12 weeks, defined as long Covid. Sick leave due to Covid-19 is an unexplored area, and increased knowledge is essential for a national and global perspective. The results from this study show that Covid-19 places a large burden not only on affected people and the health-care system, but also on the health insurance system. A large proportion of people with Covid-19 had a protracted course of sick leave, in both the present study and a published preprint [12], and a long-term perspective is needed for these patients. The actual prevalence of long Covid is unclear both globally and in Sweden [16], but the present study suggests that it affects a substantial proportion of patients, as 13% of the people with sickness benefit due to Covid-19 were on sick leave for long Covid. To the best of our knowledge, this is the first study to estimate long Covid and its consequences from sick-leave data. In a preprint study [12] using self-reported symptoms as a measure of long Covid, the majority reported that their ability to work was reduced for several months after Covid-19, but 27% were working as before. This indicates that the actual prevalence of long Covid is even higher in Sweden than the number on sick leave for long Covid presented in this study.

The need for inpatient care due to Covid-19 was a significant predictor of sick leave ≥1 month, and it increased by more than three-fold the odds of needing sick leave for long Covid compared with no inpatient care due to Covid-19. It is likely that a patient who requires extensive care due to critical Covid-19 illness will have restrictions in function for a long time [19]. Five years after being treated at an intensive care unit for acute respiratory distress syndrome, people still had decreased levels of physical function [20]. In line with the present findings, hospitalisation has also been shown to predict protracted symptoms after Covid-19 [11], and long-term symptoms exist in both hospitalised and non-hospitalised Covid-19 patients in a preprint study [13]. People suffering from long Covid seem to be a heterogeneous group [13], including both those who have been critically ill in the acute phase and those with non-critical illness, and it may be speculated that the consequences of Covid-19 differ in the two groups. Possible sex differences are one factor to consider.

Prior studies have repeatedly reported that men have more severe Covid-19, with higher mortality, a need for more medical care, and worse outcomes [21]. In this study, in line with previous research, the patients who received inpatient care due to Covid-19 were mostly men (64%). Furthermore, the proportion of men was significantly higher than the proportion on women in the group on sick leave for long Covid compared with the group not on sick leave for long Covid. In addition, men had higher odds of longer sick leave for Covid-19, both in the total population and in the population receiving inpatient care due to Covid-19. However, the present study comprised all people who received sickness benefits due to Covid-19 in Sweden, of whom a clear majority (60%) were women. This can be compared with the number of confirmed cases of Covid-19 in Sweden, where women represent 52% of the cases [22]. Furthermore, in absolute numbers, there were more women than men in the long-Covid group. This is in line with previous data showing that women generally receive more sick leave in Sweden [9] and have lower odds of returning to work after various diagnoses [23, 24]. The patients receiving sick leave for long Covid seem to be a heterogeneous group, with both critical and non-critical acute phases, and it is important not to neglect any of them.

To be on sick leave for more than 28 days or more than six times in the year before having Covid-19 resulted in higher odds of sick leave ≥1 month and for long Covid in most of the analyses. This could reflect higher comorbidity in the people with prior sick leave, or a higher vulnerability for sick leave in general. Other studies have shown that there is an association between previous sick leave and sick leave after stroke [25], traumatic brain injury [26], and mental disorder [27], and the present study indicates this also true for Covid-19.

Older age predicted sick leave ≥1 month and for long Covid in most of the analyses. Older age is highly associated with worse outcome after Covid-19 [28]. Furthermore, it can be speculated that health-care providers, the SSIA, and employers all act to optimise the return to work for younger people.

The pandemic seems to strike differently depending on socioeconomic or ethical conditions, both globally [29, 30] and in Sweden [31]. Various socioeconomic factors predicted longer sick leave for Covid-19 in this study, but the results are not clear and consistent throughout the models. To get the whole picture of factors affecting post-Covid-19 outcomes, and specifically sick leave, more research is needed. Perhaps more work-related predictors have to be included, or types of persistent symptoms. Our results did not yield distinct predictions for hospitalised vs. non-hospitalised people. Perhaps other subgroups within the heterogeneous group of persons requiring long-term sick leave after Covid-19 need to be analysed in order to paint a clearer picture.

### Methodology discussion and study limitations

The present cohort included only people receiving sickness benefits, which for most people means sick leave of more than 2 weeks. In addition, compared with national age-matched data, there were notably few deaths in the cohort [32]. This could perhaps be explained by the risk of people dying before being registered for sickness benefits for Covid-19, since the medical certificate for sick leave is usually written retrospectively on referral to a different hospital ward, for patients being treated in an intensive care unit.

About one-third of the patients were classified as “Covid-19, virus undetected”, indicating insecurity in the data. Data collection was conducted in an early phase of the pandemic in Sweden, when PCR testing was limited. The authors found it most reasonable to consider the “Covid-19, virus undetected” diagnosis as a Covid-19 infection, to capture the picture of sick leave due to Covid-19. Furthermore, related diagnoses were merged into the Covid-19 sick-leave period if there was a gap shorter than 2 weeks. This decision was based on clinical experience and reasoning, to limit the risk of missing substantial sick leave due to difficulties of registration in the beginning of the pandemic. However, there is also a risk that a small number of the sick-leave periods were not due only to Covid-19. It was not possible to determine the exact prevalence of people suffering from symptoms of Covid-19 or long Covid in this study.

The ROC curves indicate that the accuracy of the regression models was low, so the predictions should be interpreted with caution.

## Conclusion

This comprehensive registry-based study showed that a substantial number of people are on sick leave due to Covid-19, and that the sick-leave period may be protracted. More than one out of ten people on sick leave were on it for more than 12 weeks (long Covid). This group of people seems to be heterogeneous and should not be neglected. Receiving inpatient care due to Covid-19, being on sick leave prior to Covid-19, and older age all seemed to be associated with longer sick leave. The present findings are important for the health care and health insurance authorities.

## Supplementary Information

**Additional file 1.**

**Additional file 2.**

## Data Availability

The datasets used and/or analysed during the current study are available from the corresponding author on reasonable request. They are not publicly available, in accordance with the Ethics Review Authority. The study protocol and statistical analysis plan are available at https://www.researchweb.org/is/vgr/project/274476.

## Abbreviations

SSIA: Swedish Social Insurance Agency
ROC: Receiver Operating Characteristics
SD: Standard deviations
IQR: Interquartile range
OR: Odds ratio
CI: Confidence interval
