# Supplementary material for: Patterns and predictors of sick leave after Covid-19 and long Covid in a national Swedish cohort
Source: BMC Public Health. 2021 May 31;21:1023. doi: 10.1186/s12889-021-11013-2 (PMC8164957; doi:10.1186/s12889-021-11013-2)
Supplement: Supplementary file 1 — Additional file 1. [file 12889_2021_11013_MOESM1_ESM.docx]

Additional table 1. Diagnoses related to Covid-19

| Diagnoses related to Covid-19 | ICD codes |
| --- | --- |
| Acute bronchitis | J20.9 |
| Acute lower respiratory infection | J22, J22.9 |
| Acute laryngopharyngitis/acute upper respiratory infection | J06, J06.8, J06.9 |
| Acute laryngitis | J04.0 |
| Asthma | J45, J45.9 |
| Acute sinusitis | J01.9 |
| Influenza due to unidentified influenza virus | J11, J11.8 |
| Chronic tonsillitis | J35.0 |
| Virus infection | B34.2, B34.8, B34.9, B34-P |
| Sequelae of infectious and parasitic disease* | B94 |
| Postviral fatigue syndrome* | G93.3 |
| Fever | R50.9 |
| Malaise and fatigue | R53, R53.9 |
| Headache | R51.9 |
| Haemoptysis | R04.2 |
| Deep vein thrombosis* | Z86.7B |
| Family history of asthma and other chronic lower respiratory disease | Z82.5 |
| Nonrheumatic aortic (valve) insufficiency* | I35.1 |
| Anxiety disorder* | F41.0, F41.9, F41.9P |
| Reaction to stress* | F43.0, F43.8, F43.8A |
| Depressive disorder* | F32.1, F32.9 |

* = related to Covid-19 only if it was registered after the Covid-19 diagnosis (U07). Abbreviations: ICD: International Statistical Classification of Diseases.
