# Supplementary material for: Patterns and predictors of sick leave after Covid-19 and long Covid in a national Swedish cohort
Source: BMC Public Health. 2021 May 31;21:1023. doi: 10.1186/s12889-021-11013-2 (PMC8164957; doi:10.1186/s12889-021-11013-2)
Supplement: Supplementary file 2 — Additional file 2. [file 12889_2021_11013_MOESM2_ESM.docx]

Additional table 2. Primary diagnosis for inpatient care if not Covid-19

| Primary diagnosis if not Covid-19 | ICD codes |
| --- | --- |
| Cardiovascular diseases | I25, I26, I30, I48 |
| Respiratory disorders | J03, J12, J16, J17, J18, J80, J99 |
| Diseases of the abdominal organs | A09, K35, K45, K57, K80, K81, K85, K92 |
| Kidney disease | N10, N19 |
| Brain disorders | I63, G40, G93 |
| Complications during pregnancy, childbirth, and puerperium | O00, O26, O44, O47, O80, O98 |
| Viral infection | B34 |
| Symptom diagnoses | R00, R05, R06, R10, R20, R41, R50, R55 |
| Other diagnoses | T81, S82, R73, R94, D50, L03, E10, E11, F41 |

Abbreviations: ICD: International Statistical Classification of Diseases.
